# Spectrum of Associated Congenital Heart Defects in Patients with “Non-Cardiac Congenital defects at a tertiary care children hospital in Pakistan”

**DOI:** 10.12669/pjms.37.3.3604

**Published:** 2021

**Authors:** Muhammad Sohail Arshad, Muhammad Aslam, Shahnab Ahmad, Muhammad Kashif

**Affiliations:** 1 Muhammad Sohail Arshad, FCPS (Pediatric Cardiology), Department of Pediatric Cardiology, The Children’s Hospital & The Institute of Child Health, Multan, Pakistan; 2 Muhammad Aslam, FCPS (Pediatric Surgery), Department of Pediatric Surgery, The Children’s Hospital & The Institute of Child Health, Multan, Pakistan; 3 Shahnab Ahmad, FCPS (Pediatric Surgery), Department of Pediatric Surgery, The Children’s Hospital & The Institute of Child Health, Multan, Pakistan; 4 Muhammad Kashif, FCPS (Pediatric Surgery), Department of Pediatric Surgery, The Children’s Hospital & The Institute of Child Health, Multan, Pakistan

**Keywords:** Congenital heart disease, Cleft lip, Echocardiography, Patent ductus arteriosus

## Abstract

**Objectives:**

To assess the frequency and pattern of associated congenital heart disease (CHD) among patients with “non-cardiac congenital defects”.

**Methods:**

An observational study was done at Paediatric Cardiology Department, The Children’s Hospital and The Institute of Child Health, Multan, Pakistan, from December 2018 to November 2019. Children from birth to 15 years having non-cardiac congenital defects, referred for cardiac evaluation from surgical unit during the study period were enrolled. Echocardiography was done to confirm diagnosis of CHD by consultant pediatric cardiologist.

**Results:**

Out of a total of 323 cases, 176 (54.5%) were male. Out of 323 patients, 160 (49.5%) belonged to age one month to one year. Vascular malformations were the most frequent primary diagnosis among our cases, seen in 69 (21.4%) children followed by cleft lip and palate 55 (17.2%), cleft palate only 52 (16.1%), Cleft lip only 40 (12.4%) and ARM high variety 33 (10.2%). CHD was found among 42 (13.0%) children while patent ductus arteriosus (PDA) and VSD were the commonest finding seen in 14 (33.3%) and 6 (14.3%) children respectively.

**Conclusion:**

Frequency of associated CHD among patients with non-cardiac congenital defects was high (13.0%). Children with cleft lip and/or palate should be given more attention because of the high incidence of CHD in this group. Echocardiography must be advised for the timely identification of any possible type of CHD.

## INTRODUCTION

Congenital cardiac anomalies may co-exist with non-cardiac congenital malformations and, for those requiring surgical correction, there can be an anesthesia risk.^1^ This often necessitates a request for cardiovascular evaluation, including echocardiography (echo) as part of the pre-anesthesia evaluation to reduce anesthesia risk.^2^

Patients undergoing non-cardiac surgery may have conditions commonly associated with congenital heart diseases (CHD), such as children with cleft lip and palate, diaphragmatic hernia, omphalocele, tracheoesophageal fistula, skeletal anomalies, anorectal malformations and congenital vascular malformations.^3^ The presence of congenital anomalies in one system may be associated with increased incidence of congenital anomalies in other systems.^4^ It is imperative that children with such suspected cardiac anomalies or those with conditions commonly associated with CHD undergoing non-cardiac surgery should be evaluated for the presence of cardiovascular anomaly.^2^

From the studies in developing countries, the prevalence of CHD in children with cleft lip and palate ranges from 9-20%.^5^,^6^ The prevalence is higher in those with cleft palate than in those with cleft lip only. This relatively high prevalence of CHD in children with oro-facial cleft has prompted the policy in most centers of pre-anaesthetic echo for all such patients.^2^

Congenital heart defects (CHD) are considered to be the most important form of congenital defects causing high rates of morbidity and mortality among children.^7^ CHD is defined as the structural or functional heart defect which is present at the time of birth even if detection is made later on. Among children undergoing surgery for non-cardiac congenital malformations, atrial septal defect (ASD) has been noted to be the most frequent cardiac anomaly observed in 25% children followed by ventricular septal defect (VSD) in 5.8%.^8^

In a resource limited country like Pakistan, delayed diagnosis of CHD among pediatric age groups can further swell the already occurring high mortality and morbidity rates. This is why, it is vital to identify and intervene early in cases with CHD to reduce morbidity and mortality among these cases. Data from developing countries have shown that early identification and appropriate treatment among CHD cases can reduce the mortality rates from 80 to 20%.^9^ Likewise, Patients requiring non-cardiac surgery, with suspected or symptomatic structural cardiac anomalies, may have increased peri-operative risk. Following to limited availability of echocardiographic services in the country, some patients being prepared for non-cardiac surgeries are referred for pre-anaesthetic cardiovascular evaluation, including echo. The cost of echo is often high and this increases the cost of surgery. This study was conducted to describe the spectrum including frequency and pattern of associated CHD in patients with non-cardiac congenital defects. Current study is representing our 1-year experience from one of the leading government pediatric tertiary care health facility of South Punjab, Pakistan.

## METHODS

This was an observational study done at Paediatric Cardiology Department of The Children’s Hospital and The Institute of Child Health, Multan, Pakistan, from December 2018 to November 2019. Approval from Institutional Ethical Committee was obtained for this study (Ref No.10, dated: 28-10-2018).

Children from birth to 15 years, referred by Pediatric Surgery Department of the same institution to Pediatric Cardiology Unit for cardiac evaluation during the study period were enrolled. Echocardiography was done in all the referred cases to rule out CHD. Critically ill patients who could not be shifted for echo and the patients with acquired cardiac disease were not enrolled. Likewise, patients having acquired surgical problems like trauma, infections, or tumors were not enrolled. Informed consent from parents/guardians of all the enrolled participants was acquired. There was no conflict of interest as there was no financial aid involved.

A total of 323 children as per inclusion and exclusion criteria were enrolled during the study period. A special proforma was designed to record all the study data. Diagnosis of CHD was made on the basis of echocardiography findings, done by consultant pediatric cardiologist with a minimum post FCPS experience of five years. SPSS version 26.0 was used for data entry and analysis. Data was represented as frequencies and percentages.

## RESULTS

Out of a total of 323 cases, majority, 176 (54.5%) were male, 160 (49.5%) aged one month to one year and 216 (66.9%) had birth order as two or above as shown in Table-I.

**Table-I:** Overall Characteristics of Patients (n=323).

Characteristics		Number (%)
Gender	Male	176 (54.5%)
	Female	147 (45.5%)
Age	<1 month	113 (35.0%)
	>1 month to 1 Year	160 (49.5%)
	>1 to 5 years	44 (13.6%)
	>5 Years	6 (1.9%)
Birth Order	1	107 (33.1%)
	>1	216 (66.9%)

Distribution of children with respect to types of CHD and primary diagnosis is shown in Table-II. Vascular malformations were the most frequent primary diagnosis among our cases, seen in 69 (21.4%) children followed by cleft lip and palate 55 (17.2%), cleft palate only 52 (16.1%), Cleft lip only 40 (12.4%) and ARM high variety 33 (10.2%). CHD was found among 42 (13.0%) children. Among these 42 children, patent ductus arteriosus and VSD were the commonest finding seen in 14 (33.3%) and 6 (14.3%) children respectively.

**Table-II:** Distribution of Children with respect to Types of CHD and Primary Diagnosis (n=323).

Diagnosis	Omphalocele	ARM Low Variety	ARM High variety	Cleft Lip & Palate	Cleft Lip only	Cleft Palate only	Vascular Malformations	Total
Tetrology of Fallot	1		2					3
TGA with VSD with PS		1			1			2
TGA with VSD with PH			1					1
VSD		1	1	4				6
ASD		2		1	1	1		5
Complete AVSD	1			1			3	5
PDA		3	3		2	1	5	14
Tricuspid atresia	1							1
Pulmonar Stenosis		1			1	1		3
Coarctation of Aorta				1				1
Cardiac TAPVC				1				1
Total	3	8	7	8	5	3	8	42

Table-III Distribution of CHD cases in terms of gender and age groups. Acyanotic CHD were observed among majority of the cases (n=34, 81.0%).

**Table-III:** Distribution of CHD Cases (n=42).

Type of CHD	Number	Gender			Age Groups		

		Male	Female	<1 month	1 month to 1 year	>1-5 years	>5 years
Cyanotic	8	5	3	3	4	1	
Tetralogy of Fallot	3	2	1	1	1	1	
TGA with VSD with PS	2	1	1	1	1		
TGA with VSD with PH	1	1			1		
Tricuspid Atresia	1		1	1			
Cardiac TAPVC	1	1			1		
Acyanotic	34	19	15	11	17	5	1
Ventricular septal defect	6	3	3	2	3	1	
Atrial septal defect	5	3	2	2	3		
Patent ductus arteriosus	14	8	6	3	7	3	1
Pulmonary valve stenosis	3	1	2	1	2		
Coarctation of aorta	1	1			1		
Complete AVSD	5	3	2	3	1	1	
Total	42	24	18	14	21	6	1

## DISCUSSION

In the present study, frequency of associated CHD among patients with non-cardiac congenital defects was found to be 13.0%. Data from Africa evaluating pre-anesthesia echo among patients of non-cardiac surgeries found that 21.5% of them had cardiac anomalies.^2^ Oyati AI et al recorded frequency of cardiac anomalies among patients undergoing non-cardiac surgeries as 35.0% which is quite high.^8^ Data from developing countries highlight that there are increased chances of a concurrent CHD among children having congenital anomalies.^10^ In Pakistan, incidence of CHD has not been calculated in the recent years but whatever data exists, it presented the incidence of CHD as 4/1000 live births^11^,^12^ while globally, it is estimated around 8/1000 live births.^13^,^14^ So, considering these statistics, our found frequency of CHD in the present study (13.0%) is more than 32 times higher than what is usually found in the general population. So, these results from different parts of the world support our findings that we should perform echocardiography among cases undergoing surgery for non-cardiac congenital anomalies as there is an increased anaesthetic risk involved in such children.

In the present study, vascular malformations were the most frequent primary diagnosis, seen in 69 (21.4%) children followed by cleft lip and palate 55 (17.2%), cleft palate only 52 (16.1%), cleft lip only 40 (12.4%) and ARM high variety 33 (10.2%). Not much work has been done analyzing presence of CHD among children having vascular malformations. We noticed that most number of children referred to us had vascular malformations. Blei F et al from United Stats evaluating cardiac screening of infants having hemangiomas observed that out of 239 children evaluated, 50 (21%) of them had abnormal echocardiogram while out of those 50, 39 were having ASD while 6 had VSD.^15^ Among children having CHD (n=42), we noted PDA and VSD to be the commonest finding seen in 14 (33.3%) and 6 (14.3%) children respectively. In the present study, 38.1% cases of diagnosed CHD had cleft lip and/or palate so these children should be given special attention during pre-surgery evaluation for the identification of any possible CHD. Echocardiography can be a worthy option among children who are having cleft lip and/or palate prior to any surgical corrections and it might be vital to correct the heart defect before going for the cleft lip and/or palate surgery.^16^

Oyati AI et al anlalyzing echocardiographic findings among children having non-cardiac congenital anomalies and undergoing surgeries noted that cleft lip or palate were the most frequent surgical anomalies seen in 38.3% children followed by ano-rectal malformations seen in 26.7%.^8^ Sun T et al from Eastern China analyzing malformations linked with various kinds of orofacial clefts observed that out of 2180 children, 657 (30.1%) were having some kind of congenital anomaly. However, CHD formed major chunk of those congenital anomalies accounting for 296/657 (45.1%) cases.^17^ They also noted ASD to be the commonest CHD finding followed by VSD. Whereas, PDA was the 3^rd^ most frequent types of CHD. Acyanotic types of CHD formed the major chunk of CHD in the present study, accounting for 81.0% cases. These findings are consistent with the previous findings where acyanotic CHD has been observed to be the predominant portion of all cases of CHD among cases evaluated through echocardiography for non-cardiac surgery evaluation.^18^,^19^

### Limitations of the study:

It is a single center study of only one-year period. Larger, multi center studies over a longer period of time may reflect even better insight into the prevalence of CHD amongst patients with non-cardiac surgical problems. Furthermore, studies should also be planned to see the outcome of such cardiac patients after their non-cardiac surgical treatment.

## CONCLUSIONS

Frequency of associated CHD among patients with non-cardiac congenital defects was high (13.0%). Children with cleft lips and/or palate should be given more attention as they have got the highest incidence of CHD. Echocardiography must be advised for the timely identification of any possible type of CHD in all the patients presenting with non-cardiac congenital defects.

### Authors Contribution:

**MSA:** Conceived, responsible for data’s authenticity and integrity.

**MA:** Data collection, literature review.

**SA:** Data collection, data interpretation, discussion.

**MK:** data analysis, drafting.
